# Low-FODMAP Diet Improves Irritable Bowel Syndrome Symptoms: A Meta-Analysis

**DOI:** 10.3390/nu9090940

**Published:** 2017-08-26

**Authors:** Emma Altobelli, Valerio Del Negro, Paolo Matteo Angeletti, Giovanni Latella

**Affiliations:** 1 Department of Life, Health and Environmental Sciences, Epidemiology and Biostatistics Unit, University of L’Aquila, 67100 L’Aquila, Italy; paolomatteoangeletti@gmail.com; 2 Department of Life, Health and Environmental Sciences, Gastroenterology Unit, University of L’Aquila, 67100 L’Aquila, Italy; valerio.delnegro@gmail.com (V.D.N.); giovanni.latella@cc.univaq.it (G.L.)

**Keywords:** irritable bowel syndrome, nutrition, meta-analysis, epidemiology

## Abstract

Irritable bowel syndrome (IBS) affects 7–15% of the general population. A recently devised dietary approach consists of restricting foods with highly fermentable oligo-, di-, and monosaccharides, and polyols (FODMAPs), which can trigger and/or exacerbate IBS symptoms. The aim of this study is to use meta-analysis to provide an update on the randomised control trials (RCTs) and cohort studies, and examine them separately in relation to diet type. Papers were selected using the Preferred Reporting Items for Systematic Reviews and Meta-Analyses (PRISMA) flowchart. Cohen’s *d* and odds ratios were used as a measure of effect size for RCTs. A random effects model was used to account for different sources of variation among studies. Heterogeneity was assessed using Q statistics, *I*^2^, Tau, and Tau^2^. Publication bias was analysed and represented by a funnel plot, and funnel plot symmetry was assessed with Egger’s test. The results showed that in the RCTs, the patients receiving a low-FODMAP diet experienced a statistically significant pain and bloating reduction compared with those receiving a traditional diet; as regards to stool consistency, there was no significant difference between treatments. A significant reduction in abdominal pain and bloating were described by patients receiving a low-FODMAP diet compared with those receiving a high-FODMAP diet. In cohort studies, pain and bloating were significantly reduced after treatment compared with the baseline diet. We conclude that there is evidence that a low-FODMAP diet could have a favourable impact on IBS symptoms, especially abdominal pain and bloating. However, it remains to be demonstrated whether a low-FODMAP diet is superior to conventional IBS diets, especially in the long term.

## 1. Introduction

Irritable bowel syndrome (IBS), inflammatory bowel disease (IBD), and colorectal cancer (CRC) are chronic intestinal conditions whose high incidence and prevalence make them major healthcare problems [1,2,3,4,5]. IBS affects 7–15% of the general population [4,5]. It is twice as frequent in women [6] and is diagnosed more often in patients less than 50 years of age [7]. It is characterised by recurrent episodes of functional gastrointestinal symptoms whose pathophysiological mechanisms are not completely clear [8]. The most common symptoms include abdominal pain, bloating, constipation, and/or diarrhoea [8]. IBS negatively impacts quality of life and causes a substantial burden on healthcare resources [9,10]. Like the clinical phenotypes, the pathophysiological mechanisms underlying the syndrome are heterogeneous and not fully understood [11]. However, there is evidence that IBS may result from a combination of gastrointestinal motility changes, visceral hypersensitivity, low-grade inflammation, altered microbiota, and food components [12,13,14,15]. Due to the diversity of IBS symptoms and their considerable variability over time, a wide range of pharmacological treatments are employed which often only target the primary symptom; thus, when multiple symptoms are present, the treatments administered are often inadequate. This has led to the investigation of use of dietary therapies as a treatment option. Food is therefore a central and constant issue for patients with IBS. Up to 70% of IBS patients associate symptom onset or exacerbation with certain foods [16,17,18,19]. However, avoiding foods such as dairy products, wheat, citrus fruit, caffeine, and alcohol often results in negligible symptom improvement [4,18,20]. Current dietary advice for IBS patients includes regularly scheduled meals, a reduction in fibre intake, elimination of lactose-containing foods, avoidance of trigger foods, which are most commonly dairy products, wheat, and fructose, avoidance of gas-producing foods such as beans, cabbage, and onions, and limitations on caffeine, alcohol, and fatty foods [18,21]. Elimination of lactose-containing foods is still highly controversial, as this is not required by all patients. Some IBS patients have shown good lactose tolerance. A recently devised dietary approach consists of restricting foods with highly fermentable oligo-, di-, and monosaccharides, as well as polyols (FODMAPs), which can trigger and/or exacerbate IBS symptoms [22,23]. FODMAPs are osmotically active short-chain carbohydrates (SCCs) that are poorly absorbed and rapidly fermented by gut bacteria [24,25,26]. Increased intraluminal water volume, due to osmotic activity and gas production from their fermentation, causes intestinal luminal distension and induces gastrointestinal symptoms in susceptible individuals [27]. Furthermore, FODMAPs also appear to be involved in symptom generation through direct and indirect effects on gut microbiota, gut barrier, immune response, and visceral sensation [28]. It has been reported that a low-FODMAP diet can have a positive impact on IBS symptoms [26,29,30,31,32].

The main mechanism of action of low-FODMAP diets is thought to be a reduction in small intestinal absorption of osmotically active SCCs, resulting in diminished intestinal water content and downstream effects on colonic fermentation and gas production [33,34]. Recent studies have reported that, compared to baseline, low-FODMAP diets reduce the serum levels of proinflammatory interleukins (ILs) IL-6 and IL-8, the levels of faecal bacteria (*Actinobacteria*, *Bifidobacterium* and *Faecalibacterium prausnitzii*), faecal total short-chain fatty acids (SCFAs), and n-butyric acid [35,36,37,38].

The response to a low-FODMAP diet may be associated with factors related to patient demographics, microbiome composition and metabolism, and IBS subtype; however, there are no large-scale studies of its predictors [27,39]. The ability to predict responses would not only enable the ability to streamline resources and improve clinical results, but also provide insights into pathogenic mechanisms.

A recent meta-analysis, including data up to March 2015 [40], completed randomised control trials (RCTs) stratified by outcome, but the study did not divide diets by FODMAP type. The meta-analysis in this study provides an update on the RCTs and cohort studies that have been published in the intervening period and examines them separately in relation to diet type. In particular, it compares: (i) low-FODMAP diets and traditional IBS diets in RCTs; (ii) low- and high-FODMAP diets in RCTs; and (iii) baseline and post-treatment data in cohort studies of patients receiving a low-FODMAP diet.

## 2. Materials and Methods

**Figure 1:**
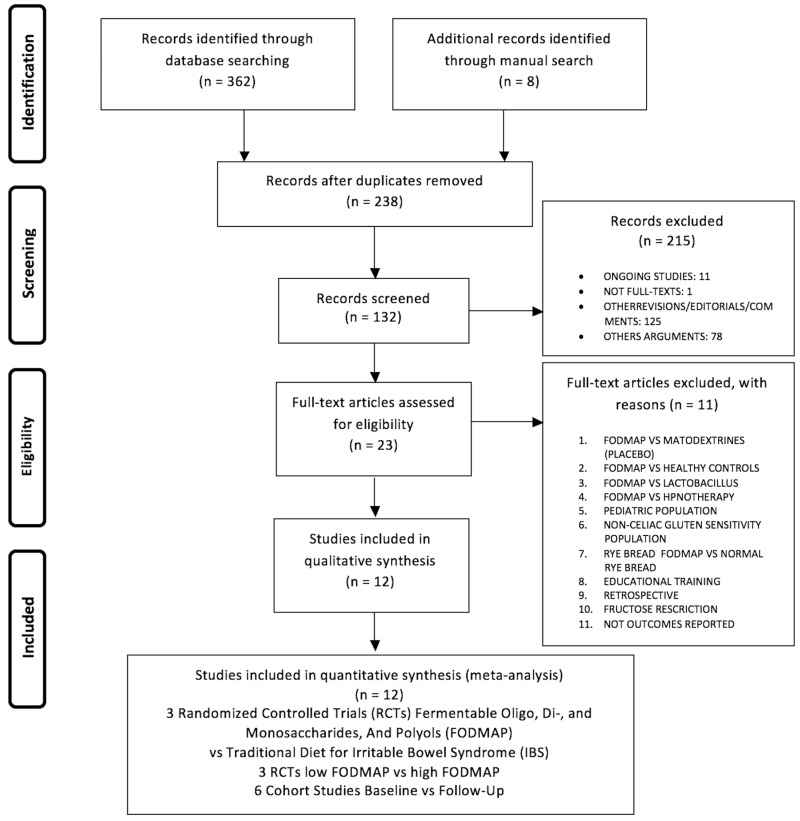
Flow chart search strategy.

The papers to be included in the meta-analysis were sought in the MEDLINE, EMBASE, Scopus, Clinicaltrials.gov, Web of Science, and Cochrane Library databases in March 2017. The search terms used were: FODMAP OR FODMAPS OR fermentable, poorly absorbed, short-chain carbohydrates, OR fermentable oligosaccharides, disaccharides, monosaccharides and polyols and (FODMAP OR FODMAPs OR fermentable, poorly absorbed, short-chain carbohydrates, OR fermentable oligosaccharides, disaccharides, monosaccharides and polyols) AND (Irritable Bowel Syndromes OR Syndrome, Irritable Bowel OR Syndromes, Irritable Bowel) OR (Colon, Irritable OR Irritable Colon) OR (Colitis, Mucous OR Colitides, Mucous OR Mucous Colitides OR Mucous Colitis). Papers were selected using the Preferred Reporting Items for Systematic Reviews and Meta-Analyses (PRISMA) flowchart (Figure 1) and the PRISMA checklist (Table S1) [41].

A manual search of possible references of interest was also performed. Only studies published in English over the previous 10 years were considered. The papers were selected by three independent reviewers (P.M.A., V.D.N., and G.L.); a methodologist (E.A.) resolved any disagreements. The study included clinical investigations involving the effect of a FODMAP diet on IBS patients. In particular, we assessed RCTs comparing a low-FODMAP diet with a traditional IBS diet, and a low-FODMAP diet with a high-FODMAP diet; cohort studies examining the effect of a low-FODMAP diet, comparing baseline with the follow-up, were also included. Outcomes evaluated were abdominal pain and bloating, which were assessed in all three study types. Since stool consistency and frequency were evaluated in all RCTs comparing FODMAP and traditional diets, these outcomes were also included.

Bias was assessed using the Cochrane Collaboration tool for assessing risk of bias [42] and the Newcastle-Ottawa scale for cohort studies (Tables S2 and S3) [43].

### Statistical Analysis

Cohen’s *d*, with 95% confidence interval (CI) and *p*-value, was used as a measure of effect size. Odds ratios (ORs), with 95% CI and *p*-value, were used as a measure of effect size for the RCTs.

Effect sizes were pooled across studies to obtain an overall effect size. A random effects model was used to account for different sources of variation among studies. Heterogeneity was assessed using Q statistics, *I*^2^, Tau, and Tau^2^. The stability of study findings was checked with moderator analysis.

Publication bias was analyzed and represented by a funnel plot, and funnel plot symmetry was assessed with Egger’s test [44]. Finally, publication bias was checked using the trim and fill procedure; we used Rosenthal’s estimator and fail-safe number to analyze publication bias [45]. PROMETA 3 software (IDo Statistics-Internovi, Cesena, Italy) was used.

## 3. Results

The search found 362 records in the databases and eight records through the manual search. After the removal of 132 duplicates there remained 238 papers; of these, 215 were excluded for different reasons (Figure 1). In the second phase of the PRISMA flow-chart, full-text articles were identified for eligibility; of these, 11 were excluded for the following reasons: three compared the FODMAP to a placebo [35], lactobacillus [46], or hypnotherapy [47], one considered healthy controls versus IBD patients [48]; one involved a paediatric population [39]; one administered the FODMAP to non-celiac gluten-sensitive patients [49]; one included interventions that only regarded two different types of rye bread (normal versus low-FODMAP rye bread) [31]; one study reviewed two different types of educational training [50]; one was a retrospective study [51]; one regarded fructose restriction [27]; and finally one did not include any outcomes of interest for our study [52]. This left six RCTs, of which three compared the traditional IBS diet to the low-FODMAP diet [29,30,36] and three compared the low- and high-FODMAP diets [25,26,53] (Table 1). Six cohort studies [54,55,56,57,58] compared patients’ conditions at baseline and after administration of the low-FODMAP diet (Table 2). In each meta-analysis, sensitivity analysis indicated that the meta-analytical findings were stable.

### 3.1. Low-FODMAP Diet Versus Traditional IBS Diet

The primary studies (*k* = 3 RCTs) by Bohn [29], Eswaran [30], and Staudacher [36] compared groups of IBS patients receiving a low-FODMAP diet to those receiving a traditional diet. These studies examined four outcomes: reduction of abdominal pain, reduction of abdominal bloating, increase of stool consistency, and reduction of stool frequency. Their main features are reported in Table 1.

#### 3.1.1. Abdominal Pain

**Figure 2:**
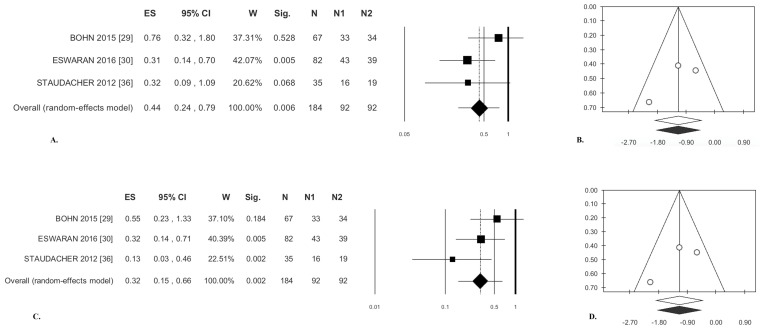
Low-FODMAP diet versus traditional IBS diet. Abdominal pain: (**A**) forest plot and (**B**) funnel plot. Bloating: (**C**) forest plot and (**D**) funnel plot.

The present meta-analysis demonstrates that the patients receiving a low-FODMAP diet experienced a statistically significant pain reduction compared to those receiving a traditional diet. The overall effect size was odds ratio (OR) = 0.44 (Table 3); there was no statistical heterogeneity (Table 3, Figure 2A). Publication bias analysis did not highlight any differences between observed and estimated values (zero trimmed studies) (Figure 2B). Egger’s test was not statistically significant (Table 3).

#### 3.1.2. Bloating

Patients managed with a low-FODMAP diet experienced significant bloating reduction compared with those receiving a traditional diet, OR = 0.32 (Table 3), and there was no significant heterogeneity (Table 3, Figure 2C). Analysis of publication bias by the trim and fill method did not lead to the exclusion of any paper (Figure 2D). Egger’s test was not significant (Table 3).

#### 3.1.3. Stool Consistency

**Figure 3:**
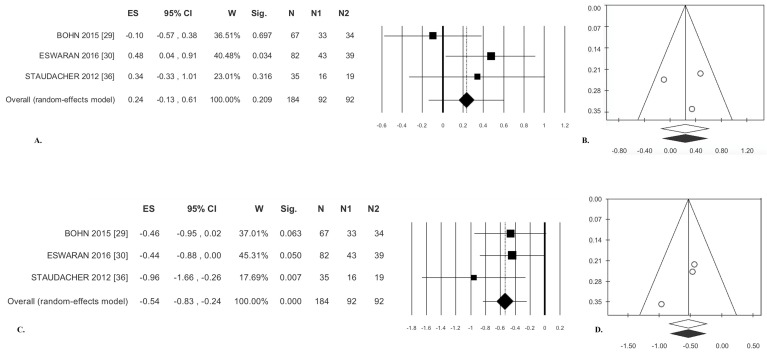
Low-FODMAP diet versus traditional IBS diet. Stool consistency: (**A**) forest plot and (**B**) funnel plot. Stool frequency: (**C**) forest plot and (**D**) funnel plot.

There was no significant difference between treatments (effect size (ES) = 0.24, Table 3); statistical heterogeneity was moderate but not significant (Figure 3A). Analysis of publication bias with the trim and fill method failed to exclude any paper (Figure 3B). Egger’s test was not significant (Table 3).

#### 3.1.4. Stool Frequency

There was a significant difference between treatments for this outcome (ES = −0.54; *p* < 0.001). There was no statistical heterogeneity (Table 3, Figure 3C). Analysis of publication bias with the trim and fill method failed to exclude any paper (Figure 3D). Egger’s test was not significant (Table 3).

### 3.2. Low-FODMAP Diet vs. Medium/High-FODMAP Diet

The primary studies (*k* = 3 RCTs) compared patients managed with a low-FODMAP diet and patients receiving a high/medium-FODMAP diet [25,26,53]. Their main characteristics are listed in Table 1. This set of studies examined two outcomes: reduction of abdominal pain and of bloating.

#### 3.2.1. Abdominal Pain

**Figure 4:**
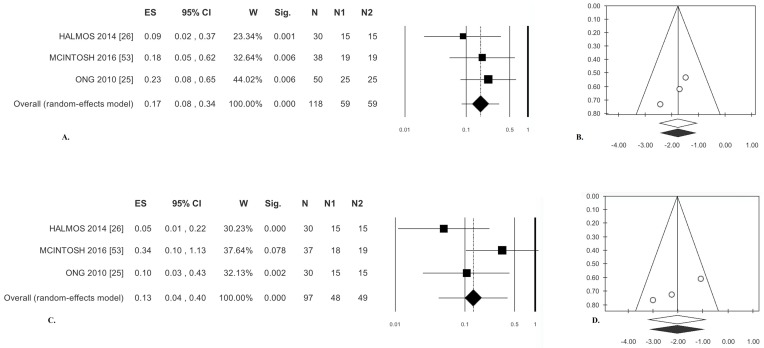
Low-FODMAP diet versus medium/high-FODMAP. Abdominal pain: (**A**) forest plot and (**B**) funnel plot. Bloating: (**C**) forest plot and (**D**) funnel plot.

Significantly reduced abdominal pain was described by patients receiving a low-FODMAP diet compared with those receiving a high-FODMAP diet (OR = 0.17). There was no statistical heterogeneity (Table 3). Analysis of publication bias with the trim and fill method failed to exclude any paper (Figure 4B). Finally, Egger’s test was not significant (Table 3).

#### 3.2.2. Bloating

The patients receiving a low-FODMAP diet reported a significant reduction of bloating compared with those given a high-FODMAP diet (OR = 0.13); statistical heterogeneity was moderate but not significant (Table 3, Figure 4C). Analysis of publication bias by the trim and fill method did not lead to the exclusion of any paper (Figure 4D). Egger’s test was not significant (Table 3).

### 3.3. Cohort Studies

The primary studies (*k* = 6) compared baseline versus post-treatment data in patients treated with a low-FODMAP diet [34,54,55,56,57,58] (Table 2). Two outcomes were assessed in this set: reduction of abdominal pain and reduction of bloating. Meta-regressions were performed for both outcomes using gender, age, and year of publication.

#### 3.3.1. Abdominal Pain

**Figure 5:**
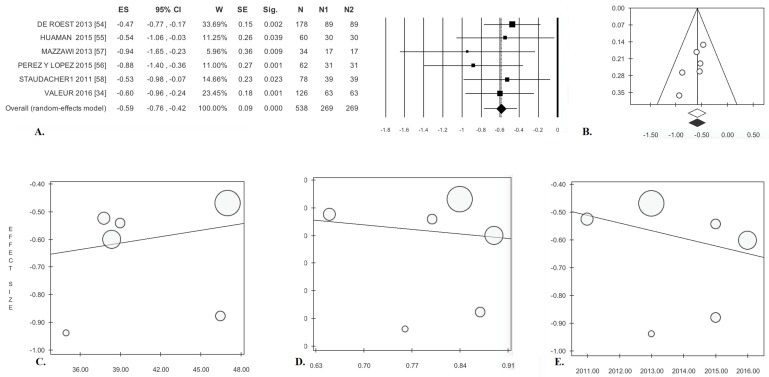
Low-FODMAP diet in cohort studies. Abdominal pain: (**A**) forest plot and (**B**) funnel plot. Meta-regression: (**C**) mean age, (**D**) gender, and (**E**) publication year.

Pain after treatment was significantly reduced compared with baseline in these patients (ES = −0.59). There was no statistical heterogeneity (Table 3, Figure 5A). Analysis of publication bias by the trim and fill method did not result in the exclusion of any paper (Figure 5B). Egger’s test was not significant (Table 3). The meta-regression lines for age (*p* = 0.652), gender (*p* = 0.817), and year of publication (*p* = 0.543) were not significant (Figure 5C–E).

#### 3.3.2. Bloating

**Figure 6:**
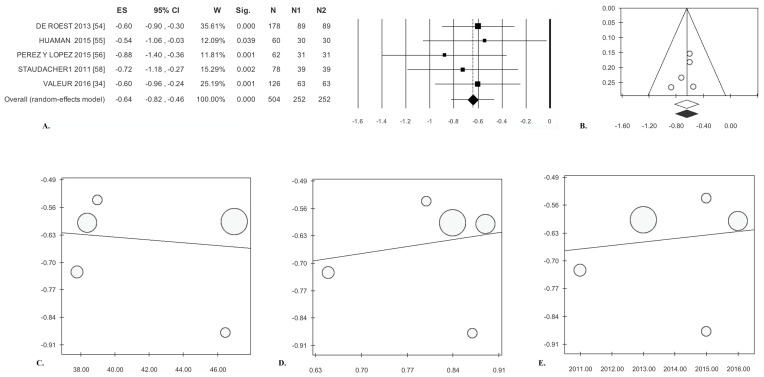
Low-FODMAP diet in cohort studies. Bloating: (**A**) forest plot and (**B**) funnel plot. Meta-regression: (**C**) mean age, (**D**) gender, and (**E**) publication year.

Significantly reduced bloating was reported by patients after treatment (ES = −0.64). There was no statistical heterogeneity (Table 3, Figure 6A). Analysis of publication bias by the trim and fill method did not lead to the exclusion of any paper (Figure 6B). Finally, Egger’s test was not significant (Table 3). The meta-regression lines for age (*p* = 0.808), gender (*p* = 0.747), and year of publication (*p* = 0.804) were not significant (Figure 6C–E).

## 4. Discussion

Several clinical trials have reported that reducing high-FODMAP foods achieves adequate symptom relief in approximately 70% of IBS patients [32,59,60]. In a recent meta-analysis, Marsh et al. [40] reported the efficacy of a low-FODMAP diet on the functional gastrointestinal symptoms associated with IBS and IBD, and found a significant improvement in symptom severity and quality of life scores compared to patients receiving a normal Western diet.

The meta-analysis in this study provides an update on the RCTs and cohort studies that have been published since then, and examines them separately in relation to diet type. Our results showed that a low-FODMAP diet versus a traditional IBS diet created a statistically significant reduction in abdominal pain, bloating, and stool frequency.

Significant reductions in abdominal pain and bloating were also found in patients administered a low-FODMAP compared to those receiving a medium or a high-FODMAP diet. Similarly, analysis of the six cohort studies demonstrated a significant reduction in abdominal pain and bloating, from baseline to follow-up, in patients treated with a low-FODMAP diet. The meta-regression lines for age and gender were not significant. Overall, the results of this meta-analysis confirm those reported in the meta-analysis by Marsh et al. [40]. The first limitation of this study lies in the relatively small number of primary studies. Moreover, given the low number of primary studies, to be able to provide quality evidence, we used random effect’s model as suggested by Liberati et al. [61]. A second limitation is the lack of blinding. However, if IBS patients have a good knowledge of food and its FODMAP content, the food in their dietary treatment, or the food choices taught to them, cannot be blinded. A third limitation is the inadequate treatment duration, which does not allow for a long-term assessment. In fact, studies involving long-term follow-ups are few. In a recent retrospective study, only one third of IBS patients receiving a low-FODMAP diet were still adherent to their treatment after a median follow-up of 18 months, even though they reported reasonable symptom relief [51]. Nevertheless, a recent prospective study in the UK [62] showed that a low-FODMAP diet can be effective and nutritionally adequate up to 18 months after initial dietitian-led education. In this study, 82% of patients who concluded the short-term FODMAP restriction phase (six weeks), continued to follow an adapted FODMAP diet in which FODMAPs were gradually reintroduced, and 70% of them maintained adequate long-term symptom relief. However, it should also be highlighted that the results of the present study were not affected by statistically significant heterogeneity and publication bias is not present. A fourth limitation is the fact that, in the studies analysed in this meta-analysis, the FODMAP diet is never compared with the current standard dietary advice for IBS, as reported by the British National Institute for Health and Care Excellence (NICE) [63].

It should be emphasised that even though a low-FODMAP diet improves IBS symptoms compared with a normal diet, this does not in fact demonstrate that the low-FODMAP treatment is superior to the conventional IBS dietary intervention. Studies comparing the efficacy of a low-FODMAP diet versus proper dietary advice for IBS (NICE diet) did not show a clear-cut advantage over the low-FODMAP diet [30,50]. Furthermore, a high-FODMAP comparator diet has the potential to exaggerate symptoms in the control [32].

A recent placebo-controlled clinical trial conducted by Staudacher et al. [64] confirmed that the low-FODMAP diet reduces the relative abundance of bifidobacterial, but the co-administration of a probiotic (VLS#3) reduces the loss of the bacterial stain. The effects of IBD treatment with a low-FODMAP diet combined with probiotics need to be clarified by further clinical trials [64].

It has also been hypothesized that patients who follow this diet may be at risk of reduced intake of fiber and some micronutrients, such as calcium, iron, zinc, folate B, D vitamins, and natural antioxidants, especially in subjects with limited access to the expensive alternative items for this diet [65]. However, a prospective study [62] showed that a dietitian-led low-FODMAP diet can be nutritionally adequate for up to 18 months. Excluding the first restriction phase of six weeks, following an adapted FODMAP diet was nutritionally adequate in macronutrients, micronutrients, and energy intake, despite having a lower FODMAP content (20.6 ± 14.9 g/day) when compared to the habitual diet (29.4 ± 22.9 g/day). Another study showed that this diet does not seem to cause vitamin D and folic acid deficiencies, even in the restriction phase [66]. Although there are few studies that evaluated the nutritional adequacy of the low-FODMAP diet, it is reasonable to think that, where properly supported by an experienced dietitian, this diet can be nutritionally adequate in the long term.

## 5. Conclusions

There is evidence that a low-FODMAP diet can have a favorable impact on IBS symptoms, especially abdominal pain, bloating, and diarrhoea. However, it remains to be demonstrated whether a low-FODMAP diet is superior to conventional IBS diets, especially in the long term. In addition, further studies are needed to demonstrate whether the low-FODMAP diet is superior to the traditional IBS diet following the NICE guidelines in the long term. Long-term FODMAP depletion may entail physiological consequences on the intestinal microbiome, colonocyte metabolism, and nutritional status that should not be underestimated, and needs further investigation.

Finally, the purpose of this meta-analysis was to provide propositions to help drive future research on this topic of growing interest among researchers, and assist with designing the epidemiological studies with comparability features in order to achieve better outcomes in clinical practice.

## Figures and Tables

**Table 1:** Characteristics of the included randomised control trial (RCT) studies in the meta-analysis.

		**Patient Population**					**Year (Mean ± SD or Median)**			**% Female**			**Symptoms and Stool Characteristics**			
**Study, Year, Country**	**Duration of Follow-up**	**Assessed** **for Eligibility**	**Randomised**	**Intervention/Control**	**Drop Outs**		Low- FODMAP group	Traditional IBS group	*p*	Low- FODMAP group	Traditional IBS group	***p***	Abdom Pain *	**Bloating**	**Stool Consistency**	**Stool Frequency**
					Study group	Control group										
RCT Low-FODMAP vs. Traditional IBS Diets																
Eswaran, 2016, USA [30]	4 weeks	171	92	50/42	Study group *n* = 5	Control group *n* = 3	41.6 ± 41.7 Low- FODMAP group	43.8 ± 15.2 Traditional IBS group	(*p* = 0.49)	66.0 Low- FODMAP group	76.2 Traditional IBS group	(*p =* 0.35)	X	X	X	X
Böhn, 2015, Sweden [29]	4 weeks	84	75	38/37	Study group *n* = 5	Control group *n* = 3	44.0 Low- FODMAP group	41.0 Traditional IBS group	(*p* = 0.35)	79.0 Low- FODMAP group	84.0 Traditional IBS group	(*p =* 0.59)	X	X	X	X
Staudacher, 2012, UK [36]	4 weeks	99	41	19/22	Study group *n* = 3	Control group *n* = 3	35.2 Low- FODMAP group	35.0 Traditional IBS group	(*p* = 0.94)	63.0 Low- FODMAP group	68.0 Traditional IBS group	(*p =* 0.74)	X	X	X	X
RCT low-FODMAP vs. Medium/High FODMAP Diets																
McIntosh, 2016, Canada [53]	3 weeks	37	40	20/20	Study group *n* = 2	Control group *n* = 1	50.2 Low- FODMAP group	51.4 High- FODMAP	(*p* = NS) **	83.3 Low- FODMAP group	89.4 High- FODMAP	(*p* = NS) *	X	X	-	-
Halmos, 2014, Australia [26]	3 weeks	45	30	15/15	IBS group *n* = 7	Healthy subject group *n* = 8	41.0 IBS group	31.0 Healthy subject group	(*p* = NS) **	70.0 IBS group	75.0 Healthy subject group	(*p =* NS) **	X	X	-	-
Ong, 2010, Australia [25]	11 days	15	15	15/15	Not Reported		50.2 Low- FODMAP group	51.4 High- FODMAP	(*p* = NS) **	83.3 Low- FODMAP group	89.4 High- FODMAP	(*p* = NS) **	X	X	-	-

SD: Standard deviation, RTC: Randomized Controlled Trials, X = symptoms assessed; - = symptoms not assessed, FODMAP: Food with Highly Fermentable Oligo, Di- and Monosaccharides and Polyols IBS: Irritable Bowel Syndrome * Abdom. Pain = abdominal pain; ** NS = not significant (as reported in the included studies).

**Table 2:** Characteristics of the included cohort studies in the meta-analysis.

Study, Year, Country	Duration of Follow-Up	Assessed for Eligibility	Completed Study (No. of Patients)	Lost at Follow-Up	Years (Mean)	% Female	Symptoms and Stool Characteristics			
							Abdominal Pain	Bloating	Stool Consistency	Stool Frequency
Valeur, 2016, Norway [34]	4 weeks	97	63	34	38.4	88.9	X	X	-	-
De Roest, 2013, New Zeland [54]	15 months	192	90	102	47.0	84.4	X	X	-	-
Huaman, 2015, Spain [55]	2 months	30	24	6	40.0	79.0	X	X	-	-
Pérez y López, 2015, Mexico [56]	3 weeks	Not reported	31	0	46.4	87.0	X	X	-	-
Mazzawi, 2013, Norway [57]	3–9 months	Not reported	46	0	35.0	76.0	X	-	-	-
Staudacher, 2011, UK [58]	9 months	Not reported	43	0	37.8	65.0	X	X	-	-

X = symptoms assessed; - = symptoms not assessed.

**Table 3:** Meta-analysis results.

	Pooled Analysis			Heterogeneity					Publication Bias					
Outcome	Effect Size	CI	*p* Value	*Q*	*I*^2^	*p* Value	*T*^2^	*T*	Egger’s		Begg and Mazdumdar’s		Fail-Safe	Rosenthal
									*T*	*p* Value	*Z*	*p* Value	No.	No.
RCTs Low-FODMAP vs. Traditional IBS Diet (*k* = 3) [29,30,36]														
Abdominal Pain	0.44 (OR)	(0.26; 0.79)	0.006	2.43	17.81	0.296	0.05	0.23	−0.19	0.877	0.52	0.602	4	25
Bloating	0.32 (OR)	(0.15; 0.66)	<0.0001	1.97	0.00	0.374	0.00	0.00	−1.21	0.439	−0.52	0.602	11	25
Stool Consistency	0.24 *	(−0.13; 0.61)	0.209	3.07	34.84	0.216	0.04	0.19	−0.02	0.989	−0.52	0.602	0	25
Stool Frequency	−0.54 *	(−0.83; −0.24)	<0.0001	1.67	0.00	0.434	0.00	0.00	−5.74	0.110	−1.57	0.117	8	25
RCTs Low-FODMAP vs. Medium/High FODMAP (*k* = 3) [25,26,53]														
Abdominal Pain	0.17 (OR)	(0.08; 0.34)	<0.0001	1.14	0.00	0.567	0.00	0.00	−4.69	0.150	−1.54	0.018	17	25
Bloating	0.13 (OR)	(0.04; 0.40)	<0.0001	4.11	51.37	0.128	0.51	0.72	−8.89	0.071	−0.57	0.017	66	40
Cohort Studies (k = 6) [34,54,55,56,57,58]														
Abdominal Pain	−0.59 *	(−0.76; −0.42)	<0.0001	2.85	0.00	0.723	0.00	0.00	−2.45	0.070	−1.69	0.091	66	40
Bloating	−0.64 *	(0.82; −0.46)	<0.0001	1.20	0.00	0.878	0.00	0.00	−1.13	0.342	−0.98	0.327	59	40

CI: Confidence Interval; OR: Odds Ratio; * Cohen’s *d*.

## Supplementary Materials

The following are available online at http://www.mdpi.com/2072-6643/9/9/940/s1, Figure S1: Sensitivity analysis of RCTs with low-FODMAP vs. traditional IBS diet, Table S1: PRISMA check list, Table S2: Cochrane tool for assessing risk of bias, Table S3: Newcastle-Ottawa scale assessment for cohort studies.
