# Supplementary material for: Low-FODMAP Diet Improves Irritable Bowel Syndrome Symptoms: A Meta-Analysis
Source: Nutrients. 2017 Aug 26;9(9):940. doi: 10.3390/nu9090940 (PMC5622700; doi:10.3390/nu9090940)
Supplement: Supplementary file 1 [file nutrients-09-00940-s001.zip › nutrients-207035-suppl/Supplementary Table S2.pdf]

Supplementary Table S2. Cochrane Collaboration tool for assessing risk of bias

Study	Random sequence generation (selection bias)	Allocation concealment (selection bias)	Blinding of participants and personnel (performance bias)	Blinding of outcome assessment (detection bias) (patient-reported outcomes)	Incomplete outcome data addressed (attrition bias) (Short-term outcomes (2-6 weeks))	Incomplete outcome data addressed (attrition bias) (Longer-term outcomes (>6 weeks))	Selective reporting (reporting bias)
Low FODMAP versus Traditional IBS diet							
Eswaran 2016[30]	**Low**	**Low**	**High**	**High**	**Low**	**Not Applicable**	**Low**
Bohn 2015[29]	**Low**	**Low**	**High**	**Low**	**Low**	**Not Applicable**	**Low**
Staudacher 2012[36]	**Low**	**High**	**Unclear**	**Unclear**	**Low**	**Not Applicable**	**Low**
Low FODMAP versus High FODMAP diet							
McIntosh 2016[53]	**Low**	**Low**	**High**	**Low**	**Low**	**Not Applicable**	**Low**
Halmos 2014[26]	**Low**	**Low**	**High**	**Low**	**Low**	**Not Applicable**	**Low**
Ong 2010[25]	**Low**	**Low**	**Low**	**Low**	**Low**	**Not Applicable**	**Low**
