# Supplementary material for: Low-FODMAP Diet Improves Irritable Bowel Syndrome Symptoms: A Meta-Analysis
Source: Nutrients. 2017 Aug 26;9(9):940. doi: 10.3390/nu9090940 (PMC5622700; doi:10.3390/nu9090940)
Supplement: Supplementary file 1 [file nutrients-09-00940-s001.zip › nutrients-207035-suppl/Supplementary Table S3 .pdf]

Supplementary Table S3. New Castle-Ottawa scale assessment for cohort studies

Study ID	Selection				Comparability	Outcome		
	Representativeness of exposed cohort	Selection of the non-exposed cohort	Ascertainment of exposure	Demonstration that outcome of interest was not present at the start of the study	Comparability of the cohort on the basis of the design of analysis	Assessment of outcome	Was follow-up long enough for outcomes to occur	Adequacy of follow up cohorts
Valeur 2016[34]	***A**	**Not Applicable**	**B**	***A**	**A***	***A**	**C**	***A**
De Roest 2013[53]	***A**	**Not Applicable**	**B**	***A**	**A***	***A**	**C**	***A**
Huaman 2015[54]	***A**	**Not Applicable**	**B**	***A**	**A***	***A**	**C**	***A**
Pérez y López 2015[56]	***A**	**Not Applicable**	**B**	***A**	**A***	***A**	**C**	***A**
Mazzawi 2013[55]	***A**	**Not Applicable**	**B**	***A**	**A***	***A**	**C**	***A**
Staudacher 1 2011[57]	***A**	**Not Applicable**	**B**	***A**	**A***	***A**	**C**	***A**
