# Supplementary figures and images for: Low-FODMAP Diet Improves Irritable Bowel Syndrome Symptoms: A Meta-Analysis
Source: Nutrients. 2017 Aug 26;9(9):940. doi: 10.3390/nu9090940 (PMC5622700; doi:10.3390/nu9090940)

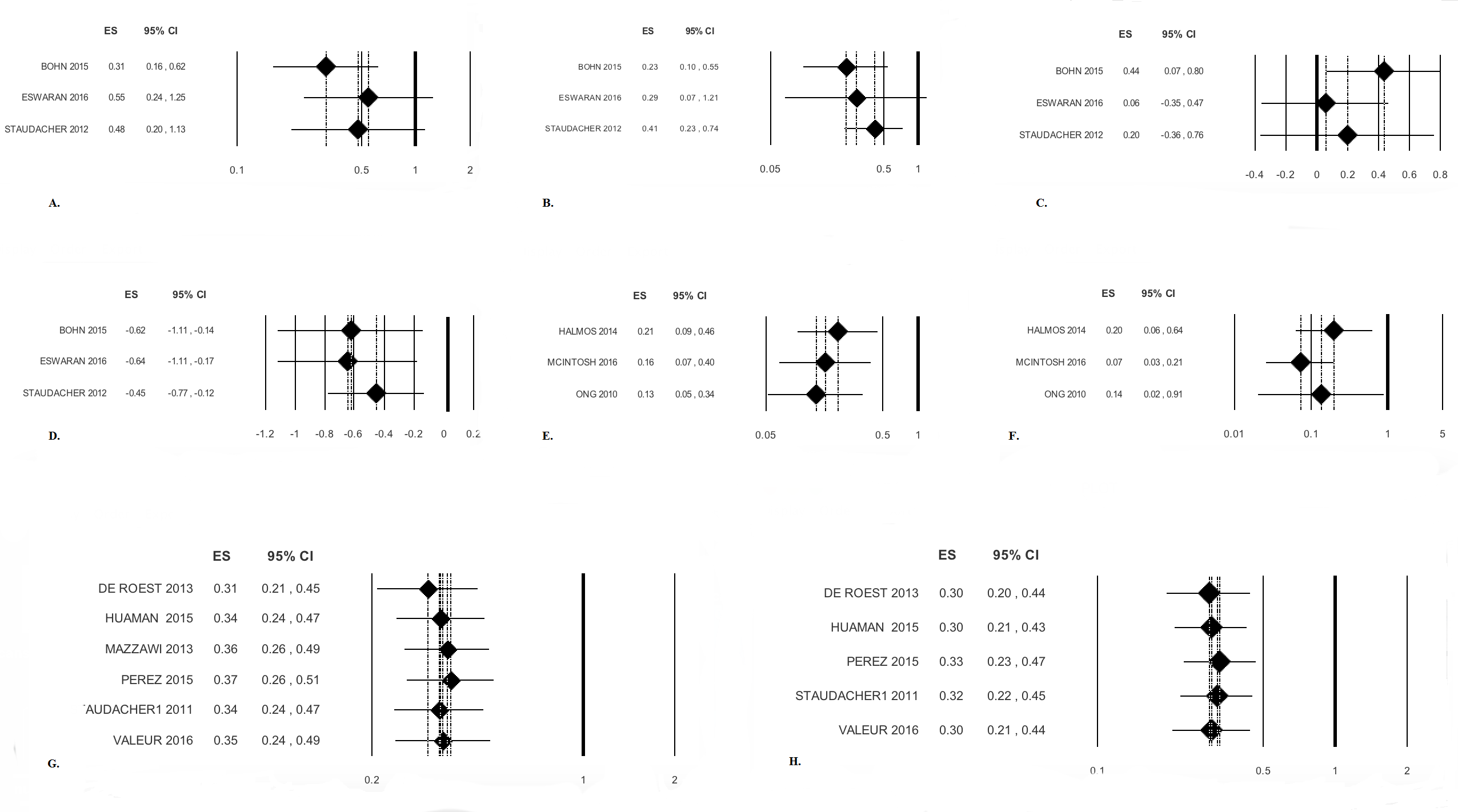

Supplement: Supplementary file 1 [file nutrients-09-00940-s001.zip › nutrients-207035-suppl/SUPPLEMENTARY FIGURE S1.tif]
